# Predicting cancer-relevant proteins using an improved molecular similarity ensemble approach

**DOI:** 10.18632/oncotarget.8716

**Published:** 2016-04-13

**Authors:** Bin Zhou, Qi Sun, De-Xin Kong

**Affiliations:** ^1^ State Key Laboratory of Agricultural Microbiology, Huazhong Agricultural University, Wuhan 430070, China; ^2^ Agricultural Bioinformatics Key Laboratory of Hubei Province, College of Informatics, Huazhong Agricultural University, Wuhan 430070, China

**Keywords:** chemoinformatics, cancer, cell line, drug development, similarity ensemble approach

## Abstract

In this study, we proposed an improved algorithm for identifying proteins relevant to cancer. The algorithm was named two-layer molecular similarity ensemble approach (TL-SEA). We applied TL-SEA to analyzing the correlation between anticancer compounds (against cell lines K562, MCF7 and A549) and active compounds against separate target proteins listed in BindingDB. Several associations between cancer types and related proteins were revealed using this chemoinformatics approach. An analysis of the literature showed that 26 of 35 predicted proteins were correlated with cancer cell proliferation, apoptosis or differentiation. Additionally, interactions between proteins in BindingDB and anticancer chemicals were also predicted. We discuss the roles of the most important predicted proteins in cancer biology and conclude that TL-SEA could be a useful tool for inferring novel proteins involved in cancer and revealing underlying molecular mechanisms.

## INTRODUCTION

In 1990, approximately 6 million people died of cancer globally, while in 2010 the number had risen to about 8 million [1]. Moreover, the incidence of cancer is increasing rapidly [2]. Current cancer treatments include surgical therapy, radiation therapy, and chemotherapy; however, these treatments also damage normal tissues. Many researchers are focused on developing targeted molecular therapies that specifically damage tumor tissues with little damage to normal tissue [3]. Comprehensive understanding of the molecular mechanisms underlying cancer is necessary for designing efficacious drugs.

The molecular mechanisms of cancer development are very complicated, and include lasting proliferation, malfunction of cell death programs, induction of angiogenesis, metastasis of cancer cells, changes of cellular energy metabolism, and evasion of immune destruction [4]. This complexity, along with a lack of reliable methods for the large-scale identification of cancer-related proteins, causes cancer progression to remain a puzzle and greatly hampers the development of effective therapies. Traditional experimental methods are not suitable for the large-scale identification of cancer-related proteins because they are both time-consuming and inefficient. Computational methods rely on systematic comparisons between the genomes of cancer cells and normal cells, using correlation analysis to search for mutated genes associated with tumorigenesis [5]. However, many of these methods only utilize DNA sequence information [6–8], and overlook protein information that is more closely related to biological pathways and phenotype.

In 2015, Chien-Hung Huang et al. developed a prediction model of cancer proteins based on proteomic data [9]. They demonstrated the accuracy of this method on two independent datasets of lung cancer and lung cancer microarray. Their prediction achieved hit ratios of 89.4% and 72.8%, respectively. Two other groups predicted cancer-related proteins as new drug targets for cancer treatment with network analyses [10, 11]. These predictions were mainly based on protein interaction networks, protein sequences, or motif information. Importantly, no cancer-related small-molecule activity data were considered in these studies. Taking into consideration the limitations of these approaches, there is a great demand for new methods to infer key proteins in cancer.

With the open-source drug discovery campaign, massive bioactivity data have been accumulated using assays ranging from phenotypes to enzymes. For example, the National Cancer Institute (NCI) has launched many programs for screening compounds against cancer cell lines and has acquired a large list of active anticancer chemicals [12]. On the other hand, BindingDB catalogues an enormous number of active compounds that act on various proteins [13].

Chemoinformatics approaches can be applied to compare anticancer compounds from the NCI database and bioactive compounds against different proteins from BindingDB, and infer proteins that are involved in the genesis and proliferation of cancer cells. The underlying assumption, named similarity principle or general neighborhood behavior, is that structurally similar molecules are likely to possess similar biological activities [14–18]. Provided that anticancer chemical a is very akin to chemical b, which is active against a protein, it is possible that a can also change the state of this protein and the anticancer effects of a depend on the same protein. The more similar molecule pairs are found, the more correct is the assumption. Therefore, the association between these proteins and cancer development can be confirmed through a systematic statistics probability analysis on massive activity data about the phenotype and the proteins.

However, the similarity between the ligand set of a protein and that of a phenotype is very weak and protein-phenotype relationships can be inferred only with very careful statistical analysis. In 2007, Michael J Keiser *et al.* proposed a Similarity Ensemble Approach (SEA) to infer relationships among receptors [19]. The SEA algorithm can be applied to two compound sets at the same level [20, 21], but is not suitable for systems at two different levels, such as active compounds against cancer cell lines and against proteins. Therefore, in this work, we proposed a modified association algorithm, named two-layer SEA (TL-SEA), and applied the algorithm to the analysis of the activity data from the NCI database and BindingDB. Three cell lines, K562, MCF7 and A549 were used as example systems. The K562 cell line was derived from the blastic phase of chronic myelogenous leukemia. It also has some characteristics of chronic leukemia and acute leukemia [22]. MCF7 and A549 were derived from human breast cancer and human lung cancer, respectively. Using TL-SEA, we attempted to infer which proteins play roles in the genesis and proliferation of these cancer cells.

## RESULTS

### Prediction of cancer-related proteins

Important cancer-related proteins were successfully predicted with our algorithm (TL-SEA) based on the existing active compounds against the three cancer cell lines and BindingDB proteins. Proteins with a smaller association value (AS score) were more likely to impact on the development of cancer. In this study, we selected the proteins with AS scores smaller than 0.03 for further analysis, resulting in a total of 35 cancer-related proteins (31, 35, and 28 proteins for K562, MCF7 and A549 cell lines, respectively; Table 1). There were 25 common proteins in the three systems. Most of the predicted proteins were human proteins or their close homologs except luciferin 4-monooxygenase of firefly. According to previously published literature, 26 of the 35 proteins are relevant to the proliferation, apoptosis, or differentiation of cancer cells. The references are listed in the last column of Table 1.

**Table 1:** List of the predicted cancer-related proteins

Protein ID*	Protein name	Source	AS score (rank)			Reference
			K562	MCF7	A549	
**P51050**	Melatonin receptor type 1B	Chicken	0.0021 (1)	0.0025 (1)	0.0025 (1)	[53]
**Q9H4B7**	Tubulin beta-1 chain	Human	0.0062 (2)	0.0064 (3)	0.0083 (4)	[54]
**P1372*6***	Tissue factor	Human	0.0065 (3)	0.0077 (6)	0.0080 (3)	[55]
**P25106**	Atypical chemokine receptor 3	Human	0.0066 (4)	0.0067 (4)	0.0093 (6)	[56]
**Q8CA95**	cAMP and cAMP-inhibited cGMP 3′,5′-cyclic phosphodiesterase 10A	Mouse	0.0083 (5)	0.0075 (5)	0.0090 (5)	
**P35355**	Prostaglandin G/H synthase 2	Rat	0.0093 (6)	0.0084 (7)	0.0103 (7)	[57]
**P60842**	Eukaryotic initiation factor 4A-I	Human	0.0103 (7)	0.0102 (8)	0.0137 (9)	[58]
**P34960**	Macrophage metalloelastase	Mouse	0.0125 (8)	0.0118 (9)	0.0149 (10)	[59]
**Q6B856**	Tubulin beta-2B chain	Bovine	0.0139 (9)	0.0124 (10)	0.0155 (11)	[60]
**P30549**	Substance-K receptor	Mouse	0.0149 (10)	0.0143 (13)	0.0175 (13)	[61]
**P06795**	Multidrug resistance protein 1B	Mouse	0.0154 (11)	0.0137 (12)	0.0176 (14)	[62]
**P52895**	Aldo-keto reductase family 1 member C2	Human	0.0160 (12)	0.0191 (19)	0.0187 (15)	[63]
**Q63008**	Sodium/iodide cotransporter	Rat	0.0172 (13)	0.0168 (15)	0.0208 (19)	[64]
**P08575**	Receptor-type tyrosine-protein phosphatase C	Human	0.0177 (14)	0.0216 (22)	0.0189 (16)	
**Q9QUK6**	Toll-like receptor 4	Mouse	0.0180 (15)	0.0201 (20)	0.0286 (28)	[65]
**P41586**	Pituitary adenylate cyclase-activating polypeptide type I receptor	Human	0.0188 (16)	0.0176 (17)	0.0171 (12)	[66]
**O02747**	Aryl hydrocarbon receptor	Rabbit	0.0198 (17)	0.0176 (16)	0.0202 (17)	[67]
**O43526**	Potassium voltage-gated channel subfamily KQT member 2	Human	0.0226 (18)	0.0261 (28)	0.0267 (24)	[68]
**Q12791**	Calcium-activated potassium channel subunit alpha-1	Human	0.0235 (19)	0.0298 (35)	0.0253 (23)	[69]
**Q27757**	Luciferin 4-monooxygenase	Firefly	0.0239 (20)	0.0153 (14)	0.0241 (20)	
**Q8R2Y0**	Monoacylglycerol lipase ABHD6	Mouse	0.0245 (21)	0.0272 (29)	-	[70]
**P49286**	Melatonin receptor type 1B	Human	0.0247 (22)	0.0245 (24)	0.0247 (22)	[53]
**P35968**	Vascular endothelial growth factor receptor 2	Human	0.0248 (23)	0.0179 (18)	0.0204 (18)	[71]
**P51787**	Potassium voltage-gated channel subfamily KQT member 1	Human	0.0268 (24)	0.0216 (21)	0.0244 (21)	
**P23097**	Collagenase 3	Rat	0.0269 (25)	0.0281 (31)	-	[72]
**P48039**	Melatonin receptor type 1A	Human	0.0270 (26)	0.0260 (27)	0.0259 (24)	[53]
**Q8TCW9**	Prokineticin receptor 1	Human	0.0278 (27)	0.0252 (26)	-	[73]
**Q13370**	cGMP-inhibited 3′, 5′-cyclic phosphodiesterase B	Human	0.0292 (28)	0.0223 (23)	0.0280 (27)	
**P48145**	Neuropeptides B/W receptor type 1	Human	0.0294 (29)	0.0284 (33)	-	[74]
**O76074**	cGMP-specific 3′,5′-cyclic phosphodiesterase	Human	0.0296 (30)	0.0282 (32)	-	[75]
**P48974**	Vasopressin V1b receptor	Rat	0.0299 (31)	0.0293 (34)	-	
**Q61614**	Endothelin-1 receptor	Mouse	-	0.0033 (2)	0.0043 (2)	
**P23907**	Major prion protein	Sheep	-	0.0134 (11)	0.0122 (8)	
**O43603**	Galanin receptor type 2	Human	-	0.0251 (25)	0.0272 (26)	[76]
**Q13698**	Voltage-dependent L-type calcium channel subunit alpha-1S	Human	-	0.0275 (30)	-	

The list was sorted by K562 significance (AS score), and then by MCF7. References regarding to the proteins related to proliferation, apoptosis, or differentiation of cancer cells were listed in the last column.

* Uniprot ID of the proteins [77].

- AS score larger than 0.03.

Among the 26 proteins, melatonin receptor type 1B occurs twice. One of them is from chicken (ranked first in all the cell lines) and the other one from human (ranked 22nd, 24th, 22nd in the 3 cell lines, respectively). BLASTP showed that these two proteins were very similar with E-Value = 7e^−150^, sequence identity = 71% and sequence cover = 100%. Melatonin receptors play an important role in cancer development [23–27], and have anticancer functions through binding with melatonin [26]. Melatonin is involved in redox processes of cells, augments natural killer cell activity, stimulates cytokine production (IL-2 and IL-6), and protects hematopoietic precursors from the toxic effect of chemotherapy and radiotherapy [27]. Studies revealed that breast cancer cell differentiation is regulated by the MT-1 signaling pathway [28, 29], while the anticancer function of melatonin is mediated by MT-1 receptor and G protein-coupled signal transduction in liver cancer cells [30]. Clinical data also showed high MT-1 expression is associated with cancer resistance in people with lower melatonin levels [31]. Melatonin may also protect against gastric cancer in mice by up-regulation of membrane receptor MT-1 and MT-2 expression [32].

The second and the third proteins in the predicted list are tubulin beta-1 chain and tissue factor (TF). Tubulin beta-1 chain is the primary component of microtubules. Microtubules play a key role in the process of mitosis [33], which is necessary for cancer cell proliferation. Thus, disruption of cell mitosis can block the increase in cancer cells. As early as 2004, there was research on microtubules as targets for anticancer drugs [34]. Similarly, TF expression in the cell surface accelerates tumor progression [35, 36]. TF accelerates malignant tumor growth, invasion, and metastasis mainly by promoting vascular endothelial growth factor (VEGF) release to regulate tumor cell angiogenesis [37]. Interestingly, the VEGF receptor 2 is ranked 23rd in the predicted protein list. Reduced TF expression can decrease cancer cell growth, and selective reduction of TF expression with mRNAi in colorectal cancer cells reduced tumor growth in mice [38]. These results have been replicated *in vitro* [39], and higher TF expression was found in primary carcinoma of the rectum, breast cancer and pancreatic cancer. Thus, TF expression is related to the invasiveness of cancer [40], and multiple experimental models have demonstrated that increasing TF expression promotes tumor growth [41].

For those proteins without direct evidence regarding their involvement in cancer development, there is a great chance that they also play important roles in cancer-related cellular pathways. Of course, this hypothesis remains to be confirmed with further studies. We analyzed protein Q8CA95 (cAMP and cAMP-inhibited cGMP 3′,5′-cyclic phosphodiesterase 10A), which is ranked 5th in all the predicted proteins and first in the proteins without direct proof in the literature. The protein hydrolyzes both cAMP and cGMP, regulating the intracellular concentration of cyclic nucleotides in the striatum [42]. As a target for signal transduction regulation, it has not been reported to have anticancereffects; however, cAMP mediates the translation of cancer cells into healthy cells [43, 44].

**Figure 1:**
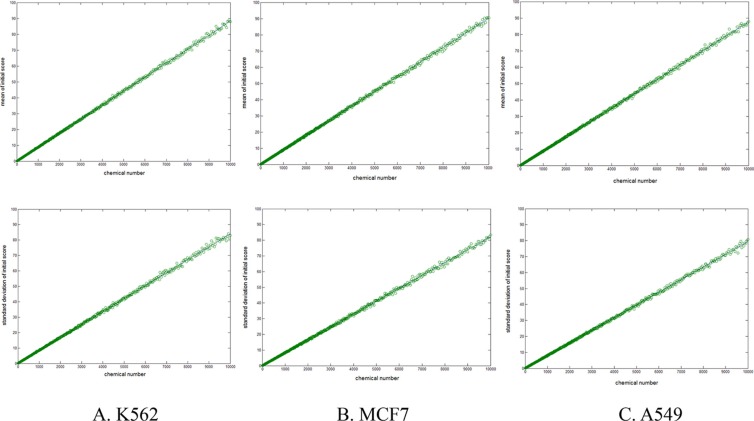
Scatter graph of the mean value (top) and standard deviation (bottom) of random initial score (*I*) with different sampling lengths (*m*, horizontal axis) (**A**) For K562 dataset. Fitting with formulae 2, 3, constant parameters were estimated (*a* = 0.0088, *b* = 0.9950, *k* = 0.0088). (**B**) For MCF7 dataset, *a* = 0.0086, *b* = 0.9952, *k* = 0.0090. (**C**) For A549 dataset, *a* = 0.0083, *b* = 0.9969, *k* = 0.0089.

In our algorithm, we needed to calculate the significance twice, *P*_Z_ and *P*_O_, for the anticancer compounds-protein association (initial score, *I*) and the cell line-protein association (original score, *P_O_*). Because *I* was summed at different length (*m*, number of active compounds against a specific protein), it was translated into comparable *Z* score with formula 4. The constants (*a, b, k*) were obtained by fitting the initial score and the number of active compounds with formulae 2 and 3 (Figure 1). The results showed a linear correlation between the initial score *I* and compound number *m*, indicating the feasibility of the SEA algorithm in such a system. As mentioned in the methods section, it is unnecessary to standardize the original score, *P_O_*.

### Analysis of chemical-protein matrices

**Figure 2:**
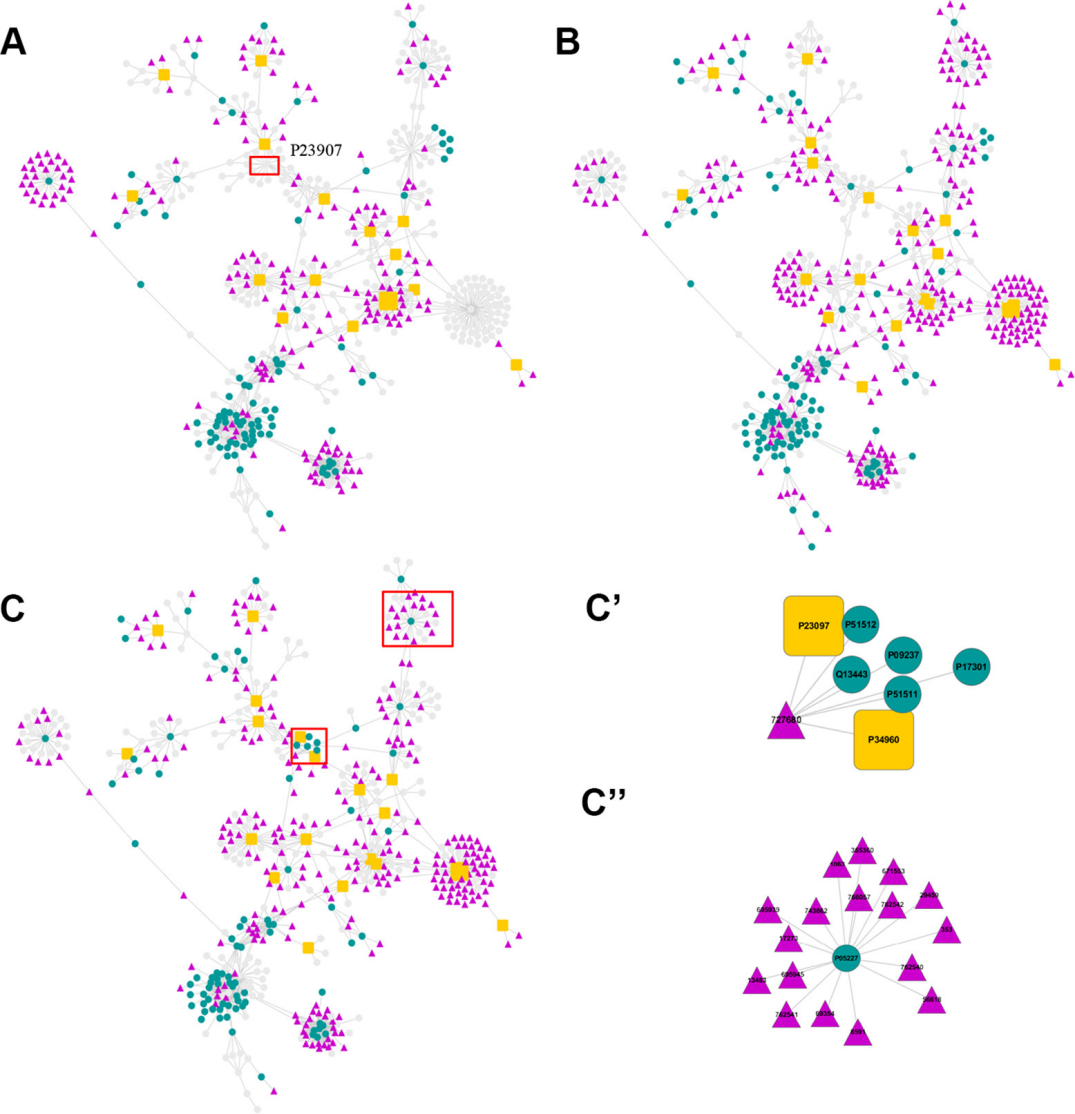
Chemical-protein association networks The NCI compounds are represented with triangle nodes. Proteins are denoted with round nodes. Among the proteins, the important ones are denoted as orange squares. (**A**) Main network for blastic phase of chronic myelogenous leukemia (K562) cell line active compounds and proteins. Gray nodes denote that it does not appear in this system. (**B**) Main network for Non Small Cell Lung cancer (A549) cell line. (**C**) Main network for breast cancer (MCF7) cell line. C') and C”) are two sub-networks extracted from the MCF7 network. See text for details.

In the process of inferring cancer-related proteins, three anticancer compounds *vs* BindingDB proteins association matrices (*P*_Z_) emerged. The matrices contained the significant scores (*P*_Z_) between the active chemicals targeting the cancer cell lines and the BindingDB proteins. *P*_Z_ can be used to deduce whether a compound can interact with a protein. Smaller *P*_Z_ indicated higher possibility of interaction. By retaining the matrix elements with *P*_Z_ less than 0.0001, the matrices were translated into three networks as shown in Figure 2 and Supplementary Figure S1. The nodes in the networks represent proteins or chemicals, while the edges denote their association. The three networks are presented with the same layout. The position of the nodes was optimized with forces according to the reciprocal of the *P*_Z_. For nodes that were missed in the cell lines, they were fade out to gray. The other nodes were highlighted with distinguishable colors. Through this way, the differences between the cell lines can be visually analyzed. For example, the major prion protein (node P23097, highlighted with the red rectangle in Figure 2A) did not existed in K562 but existed in other two cell lines. Experimental studies showed that the over-expression of P23097 failed to protect DNA fragmentation in leukemia cancer cell line but it converted TNF-sensitive cells into TNF-resistant cells in MCF7 breast cancer cell line [45–46]. Moreover, the expression of major prion protein were associated with increased lung colonization [47]. These results are consistent with our predictions.

Some of the predicted interactions in Figure 2 were reported earlier and truly existed. For example, the *P*_Z_ score between anticancer compound thapsia villosa (NCI_ ID 299934) and sarcoplasmic/endoplasmic reticulum calcium ATPase 1 (Uniprot_ID P04191, SERCA 1) was 1.500 × 10^−7^, ranking first in MCF7 dataset (see Supplementary information Table S2). This compound is indeed a potential inhibitor of the SERCA pump [48]. In addition, the *P*_Z_ score between compound GW805758X (NCI_ID 756364) and protein O14920 (inhibitor of nuclear factor kappa-B kinase subunit beta) was 1.015 × 10^−4^. Through database searching, this interaction can be found in ChEMBL (http://ebi.ac.uk/chembl, ChEMBL Assay ID: CHEMBL2007663).

We analyzed the proteins linked to more than 15 compounds in the three networks (Table 2). Protein Q61614 (Endothelin-1 receptor) linked to 59 compounds in the MCF7 dataset, ranked first in all the proteins. The AS score (*P*_O_) between this protein and MCF7 is 0.0033, indicating that this protein is a potential target for MCF7 cells. Kusuhara M et al. found that breast cancer cells can release Endothelin-1 [49]. The binding of Endothelin-1 (ET-1) to ET-1 receptor can stimulate growth of breast cancer cells by autocrine and paracrine signaling, and increased expression of ET-1, Endothelin A receptor (ETAR), and Endothelin B receptor (ETBR) in breast cancer patients lowers disease-free survival time and overall survival [50].

**Table 2:** List of the proteins linked to more than 15 anticancer compounds according to *P*_z_ < 0.0001

Protein_ID	Number of linked compounds		
	K562	MCF7	A549
Q61614	-	59*	58*
Q9H4B7	30*	27*	32*
P41586	17*	20*	25*
P07382	31	31	45
P00378	30	28	42
P11387	29	-	-
P00375	22	25	26
P49892	21	40	31
Q6Y1R5	21	41	31
P07900_P08238	18	15	-
P22102	18	22	24
P34970	18	37	28
Q8TEK3	18	40	28
O02747	17*	-	21*
Q05932	17	23	25
P17707	-	44	35
O02667	-	31	22
P23526	-	26	23
P05227	-	19	22
P15328	-	18	20
P28647	-	18	-
O00142	-	15	-
P41148	-	15	-
Q62645	-	15	17
P48544	-	-	20
P48549	-	-	19
Q01782	-	-	15

- with less than 15 linked compounds.

* predicted as a cancer related protein.

Among the 27 proteins in Table 2, only 4 proteins (Q9H4B7, P41586, Q61614 and O02747) were associated with the cell lines with an AS score (*P*_O_) less than 0.03. This result was caused by different thresholds used in the original score calculation process and in Figure 2, which illustrates the protein-compound interactions with a visual network. Only connections with *P*z < 0.0001 were retained to simplify the networks. For *P*_O_ calculation, more information was needed for association analysis. Thus, we used a threshold of *P*z < 0.01. If the threshold of the connections was changed to *P*z < 0.001, all the 11 proteins with more than 70 connections were associated with the cell lines (*P*_O_ < 0.03). The results proved the capability of our algorithm for deep data mining. That is, the association score was deduced with large numbers of weak similarities between the active compounds of the cell lines and the proteins instead of fewer but stronger similarities.

We also analyzed two sub-networks (Figure 2C', 2C”) extracted from the interaction network of MCF7 active compounds and the proteins (Figure 2C). Figure 2C' shows the predicted interactions between a MCF7 active compound (NCI_ID 727680) and 7 proteins, while Figure 2C” shows the interactions between a BindingDB protein (Uniprot_ID P05227) and 17 anticancer compounds for the MCF7 cell line. Detailed information about the subnetworks, including proteins, compounds and *P*_Z_ between them can be found in Supplementary Table S1. Full information on *P*_Z_ < 0.0001 data in MCF7 can be found in Table S2. We randomly selected 4 active compounds (NCI_ID 353, 8591, 695939, 743862) from these 17 anticancer compounds in Figure 2C” and calculated the similarity between these NCI compounds and the active compounds against the BindingDB proteins (Uniprot_ID P05227). Most of the similarities were around 0.2, except very few high similarity scores (Supplementary Table S3). This result is consistent with Keiser's research, which found that for most ligand pairs the similarity was low, ranging from 0.2 to 0.3 [19]. This result also indicates the necessity to use strict statistical algorithms in such systems and confirms our previous deductions.

## DISCUSSION

Prior methods for large-scale identification of cancer-related genes or proteins were primarily based on the discrepancies between the genomes of cancer cells and normal cells, and rarely took into consideration ligand-protein interactions. Our research employed the activity data of the chemicals targeting proteins or cancer cells in existing databases, enabling us to analyze the mechanisms underlying tumorigenesis from the perspective of chemistry. A chemoinformatics approach (TL-SEA) was proposed to compare anticancer compounds with active chemicals binding to a particular protein target. By this means, possible associations between cancer cell lines and proteins were predicted if the two groups of chemicals showed similarity. Literature searches showed that most of the high-ranked proteins were related to proliferation, apoptosis, or differentiation of cancer cells.

Additionally, a chemical-protein interaction matrix was produced, which can help explain the mechanism of the anticancer drugs and also boost the repurposing of anticancer drugs to other fields. In fact, the drugs active against the predicted proteins are also potential active compounds against cancer. This can be quantitatively measured using the TL-SEA algorithm in reverse, to calculate the association between active compounds against the predicted protein targets and NCI cell lines. The above results confirmed the effectiveness of our algorithm. Of course, further laboratory experiments are needed to validate the predicted associations/interactions. Pathway analysis and systems biology simulation can also be performed to interpret the roles of the proteins in tumorigenesis.

The applications of this chemoinformatics approach can be expanded to elucidate the molecular mechanisms of other diseases. For instance, by comparing the active compounds against a bacterium with those targeting a variety of proteins, it is possible to deduce important proteins for the growth of this bacterium. The primary mission in the post-genomic era is to illuminate the relationships among genes, proteins, diseases, pathways and chemicals at an -omics level. It is impossible to finish this work using traditional methods. Currently, a large number of small molecule activity data are becoming available to the public, such as ChEMBL and Pubchem [51, 52]. These datasets include the results of high throughput screening at the molecular level and all kinds of phenotypic activity. The relationship between the phenotypes (diseases) and proteins can be inferred using the TL-SEA algorithm proposed here.

Compared with traditional approaches, our approach is economically feasible and fast, and therefore suitable for rapid preliminary screening before further validation. Compared with conventional genome correlation analysis, TL-SEA utilizes the activity data directly, reflecting protein function in the organism. Thus, the results of TL-SEA can be interpreted more easily with pathway analysis. However, the limitations of this approach should not be ignored. The method relies on the activity data of small molecules and only applies to the diseases or proteins whose active molecules are known.

## MATERIALS AND METHODS

### General study protocol

**Figure 3:**
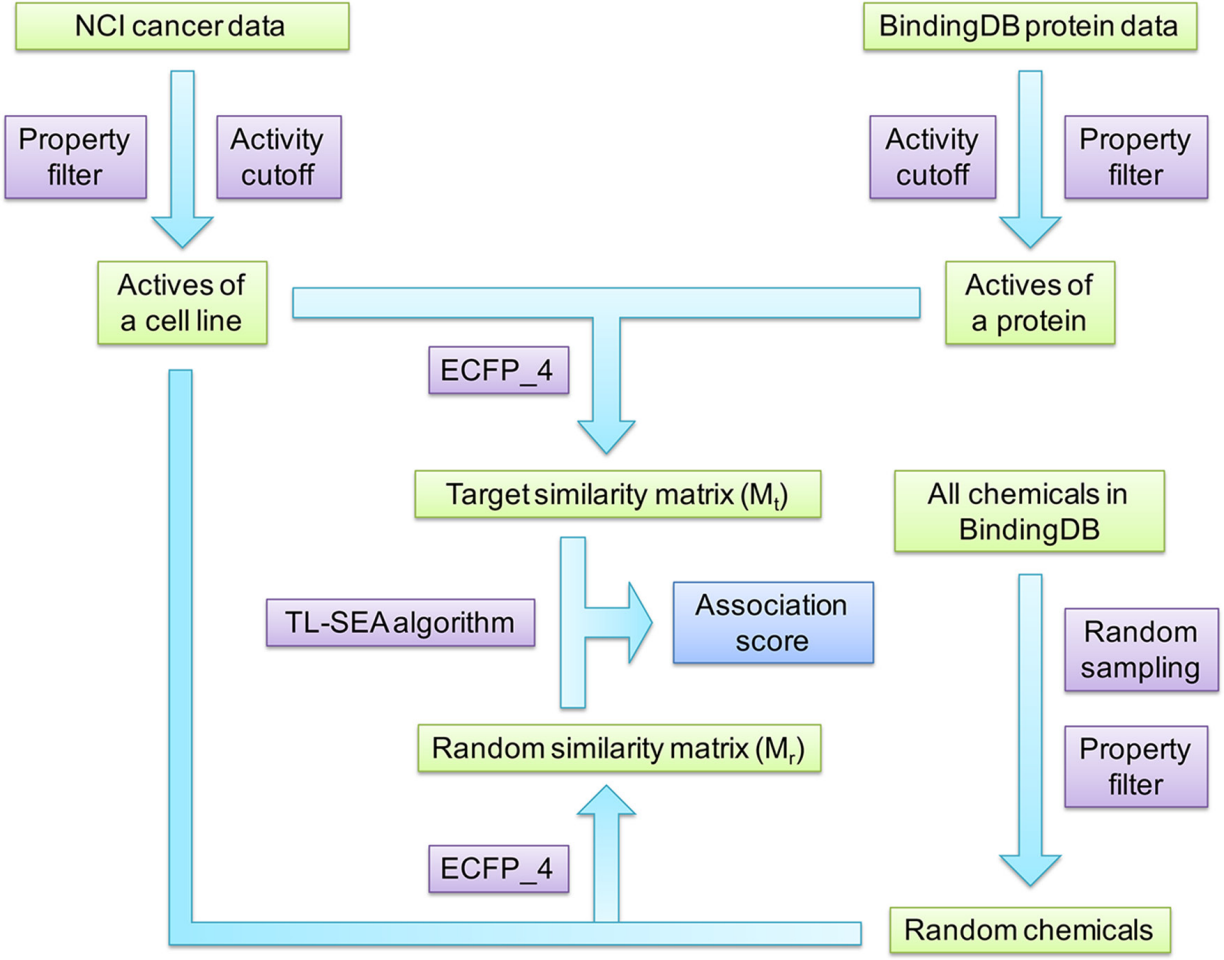
The overall protocol of this study

The overall protocol of this study is illustrated in Figure 3. Activity data and structures of small molecules against cancer cell lines and against a variety of proteins were collected from the NCI database and BindingDB. Physicochemical properties and activity thresholds were used to filter off inactive or non-druglike compounds. Then the similarity matrix (target similarity matrix) formed by these two groups of active compounds was calculated with ECFP_4 molecular fingerprint and the Tanimoto coefficient. At the same time, large numbers of chemicals satisfying aforementioned physicochemical properties were randomly sampled from BindingDB. The similarity matrix (random similarity matrix) formed by these random BindingDB chemicals and the NCI active compounds of the corresponding cell line was also calculated in the same manner. Finally, the TL-SEA algorithm was employed to compare the target similarity matrix with the random similarity matrix, and therefore give the association score (AS) between each protein and the cell line. The AS score was used to infer whether a given protein plays a role in the growth of cancer cells. Details about the association algorithm are described below.

### NCI database

Activity and structural data of cancer cell line inhibitors were downloaded from NCI website (https://wiki.nci.nih.gov/display/NCIDTPdata/Chemical+Data). NCI database uses GI_50_ (growth inhibition of 50%) as an endpoint, which is the drug concentration giving a 50% reduction in the cancer cell proliferation. According to the distribution of GI_50_ value of the cell lines, leukemia cell lines were generally more sensitive than other cell lines. Therefore, K562 leukemia cells (September 2012 release) were selected as the study material and its activity data were used for the next analysis. We also selected two other cancer cell lines, MCF7 and A549 (September 2014 release), to confirm the stability and effectiveness of our algorithm. The three cancer cell lines were analyzed separately.

Activity data sets of K562, MCF7, and A549 cell lines contained 47,497; 36,801; and 51,170 entries, respectively. 2D structures of the corresponding active compounds were extracted and linked with the activity values. The distribution of compounds' GI_50_ values, ranging from micromole to nanomole, was analyzed with the cumulative frequency plot (Supplementary Figure S2). 90% of the active compounds possessed a pGI_50_ (the negative logarithm of GI_50_ values in base 10) less than 6 (GI_50_ ≥ 10^−6^ mol/L). Therefore, compounds with pGI_50_ over 6 were defined as active, which included 3658, 3744 and 4646 compounds in the three data sets, respectively. Inactive compounds were discarded.

The distributions of molecular weight and AlogP (oil water distribution coefficient) of the anticancer active compounds were compared and analyzed (Supplementary Figure S3). AlogP thresholds were set to [2, 7], [−3, 8], and [−3, 8] for the three cell lines, respectively. Molecular weight thresholds were set to [150, 750], [200,800], and [200,800]. After property filtering, 3160, 3362, and 4150 anticancer active compounds were retained for the following analysis.

### BindingDB database

Active data against proteins were obtained from BindingDB (http://bindingdb.org/bind/index.jsp, accessed on 2 March 2014). The binding data and 2D structures of small molecules were collected. There were four types of endpoints used in BindingDB, *i.e. K*_i_, IC_50_, *K*_d_ and EC_50_. Compounds were defined as active when any of these values were smaller than 10^−6^ mol/L. To ensure the consistency of physical and chemical properties, BindingDB compounds were also filtered with the property criteria as discussed above. The final BindingDB active ligand set contained 505,600 compounds.

### Generation of the similarity matrices

A similarity matrix (M) was generated by calculating the similarities between the NCI and BindingDB active compounds. Each column of the matrix corresponds to a NCI active compound, while each row corresponds to a BindingDB active compound. The similarity was calculated with ECFP_4 molecular fingerprint and Tanimoto coefficient. ECFP is Extended-Connectivity Fingerprints based on the Morgan algorithm [78]. It is a circular topological fingerprint designed for molecular characterization, similarity calculation, and virtual screening. The diameter of a circular atom neighborhood is set to 4. Tanimoto coefficient (*S*_t_) is one of the most widely used similarity indices and is defined as *S*_t_ = C/(A + B – C). Here, A and B are the numbers of fingerprint bits of molecules A and B, and C is the number of bits coexisting in both molecules.

As background sampling, around 50,000 compounds were randomly selected from BindingDB. The compounds were filtered using physicochemical properties as described above. Similarity matrices of the randomly selected BindingDB compounds against all NCI active compounds of each cell line (M_r_) were also calculated. Three random compound sets (similarity matrices) were prepared with different property thresholds for the three NCI cell lines.

### Calculating association scores using the TL-SEA algorithm

**Figure 4:**
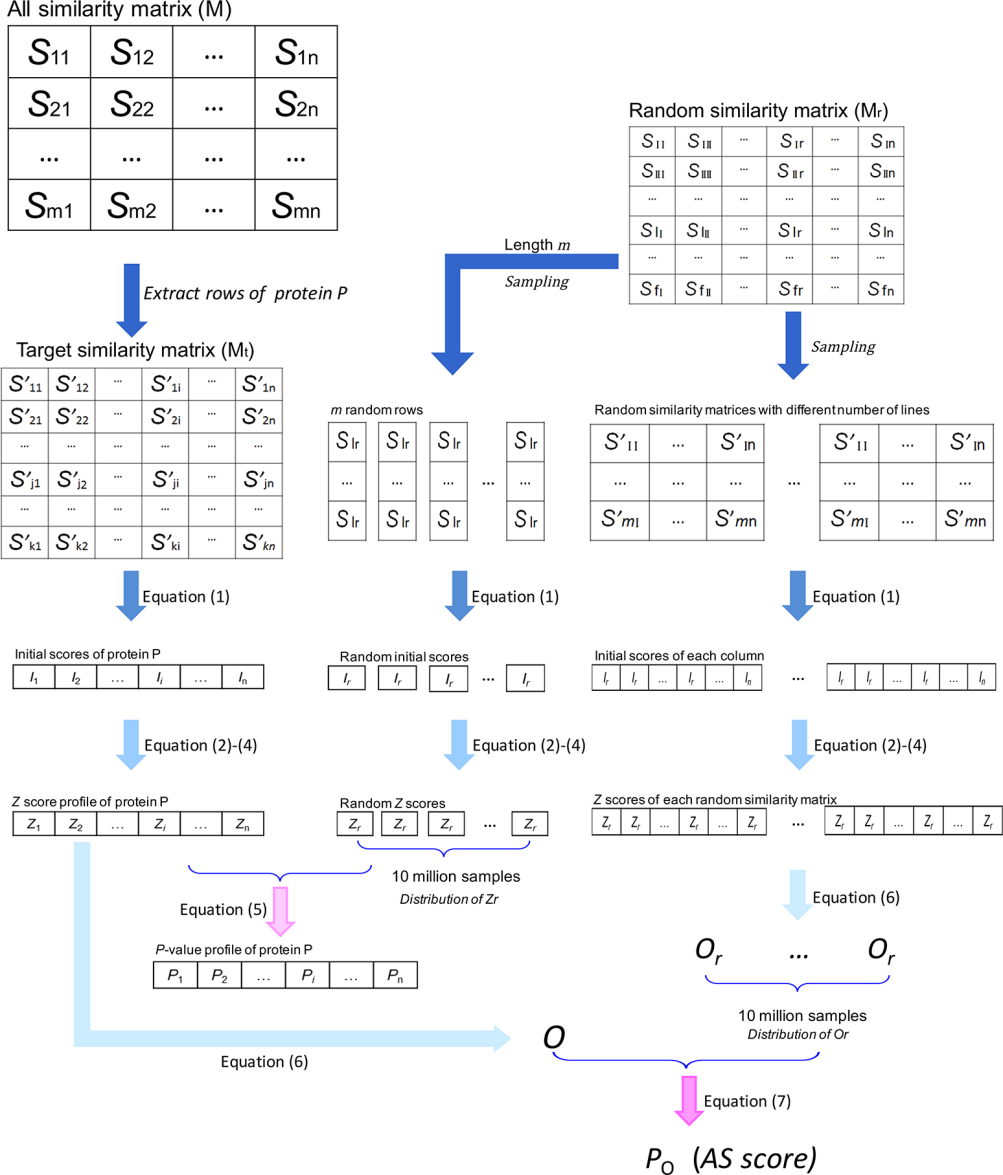
Schematic representation of the TL-SEA algorithm First, the target protein similarity matrix (M_t_) was extracted from the overall NCI-BindingDB similarity matrix (M). Then, the matrix was translated into an initial score vector. Next, the initial score vector was normalized to the Z score vector through random column sampling. Finally, the association score (AS) was calculated based on the Z score vector and another random sampling of random similarity matrixes. Here, n is the number of the active compounds of a NCI cell line. S and S' are the similarity value between NCI compound and BindingDB compound. I is the sum of the similarity values over 0.15 in the corresponding column. Refer to the text for a detailed description.

The similarity between two unrelated compound sets was usually very weak. Therefore, a sensitive association recognition algorithm with careful statistical inference was required to predict cancer-related proteins. This algorithm needed to be able to find out the implicit association of cancer cells with particular proteins using their active molecules. Based on the SEA algorithm, which was originally proposed by Keiser [19], we proposed an improved two-layer approach (TL-SEA). The protocol of this algorithm is described as follows (Figure 4).

### Extract the row vectors corresponding to a BindingDB protein active set

To analyze the association between the NCI cell line and a certain protein, the row similarity vectors corresponding to the protein's active compounds were extracted from M. The extracted row vectors composed a target sub-matrix (M_t_). Proteins with less than 10 active compounds (vectors) were discarded.

### Translate the similarity matrix M_t_ into an initial score (*I*) vector by adding up all the similarity values over a threshold in each column

The frequency histograms of the random similarity values (in M_r_) were analyzed and shown in Supplementary Figure S4. By analyzing the distribution of random similarity values in the random similarity matrices, we chose 0.15 as the threshold and used it to filter off weak similarity noise.

Then, by summing up all the similarity values over 0.15 in each column (NCI active compound *i*), the similarity matrix (M_t_) was translated in to a row vector. The element of the vector was defined as the initial score (*I*) between the protein and a particular anticancer compound.

$$I_{i}=\Sigma S_{ji}(S_{ji}>0.15)$$ (1)

Here, the summation was made over all the similarities between the active compounds against the protein (*j*) and the anticancer compound.

### Translate the initial score vector into significance score (*P*_Z_)

The initial score reflected the association between each protein and the corresponding anticancer compound. However, the initial score varied when the number of active compounds changed. For proteins with more active compounds, their initial scores were generally higher than proteins with less active compounds. Therefore, the initial score was translated into a comparable standardized score (*Z*) and significance score (*P*_Z_). This was achieved with row sampling and linear regression.

- Random sampling was conducted against all columns in the random similarity matrix (M_r_). For each randomly selected column, the sampling was performed with 2000 different lengths (*m*). The lengths were randomly selected from 1 to 10,000. The operation was repeated for 5000 times, which produced 10 million random compound sets.
- As we did in step 2, for each sampling, an initial score vector was obtained by summing up the similarity values larger than the threshold (0.15). Then, the distribution of the initial score over different sampling length (*m*) was obtained through analysis of the 10 million similarity sets (2000 × 5000). Here we used the same protocol to Keiser's [19]. First, by fitting the distribution pattern over sampling length with the following equations, constants (*a, b* and *k*) were obtained.

$$\mu_{m}=k\times m$$ (2)

$$\sigma_{m}=a\times m^{b}$$ (3)

Here, *μ_m_* and *σ_m_* are the mean and the standard deviation of *I* in each group with sampling length *m*. *a, b*, *k* are the constants of the distribution of *I* in different length *m*. Then, a standardized score (*Z*) for each initial score was calculated with the following formula:

$$Z=(I-\hat{k}\times m)/(\hat{a}\times m^{\hat{b}})$$ (4)

Here, $\hat{a},\;\hat{b},\;\hat{k},$ are the estimated values for *a*, *b* and *k*. The standardized score (*Z*) was comparable between proteins with different numbers of active compounds. Finally, the standard score was translated into a significance score (*P*_Z_) with the formula,

$$P_{Z}=N(Z_{r}\geq Z_{i})/N(Z_{r})$$ (5)

Here, *N(Z*_r_*)* is the total number of *Z* scores of the random sampling, which is equal to 10^7^. *Z*_i_ is the standardized score of the th column in the target similarity matrix of protein (M_t_). *N*(*Z*_r_ *≥ Z*_i_) is the number of *Z*_r_ which exceeds or equal *Z*_i_. *P*_Z_ is the statistical significance of the *i*th column, which is equal to the probability of getting a *Z* score no smaller than *Z*_i_ from random sampling. Therefore, the association between a cell line active compound and a certain protein can be estimated with *P*_Z_.

### Translate the standardized score (*Z*) vector into the association score (*P*_O_)

In the above steps, we compared the active compounds against specific proteins and each cell lines active compounds, producing the *Z* score rating the relationship of this protein to every anticancer compound. To analyze the association between the protein and the cell line, the *Z* score vectors were merged into a comparable association score based on random row sampling.

First, a threshold (*c*) of *Z* corresponding to an acceptable confidence level (probability *P*_Z_ = 0.01) was determined. By summing up the *Z* values not less than *c*, the original association score (*O*) of a certain protein was obtained.

O=ΣZ(Z≥c) (6)

Then, random rows were extracted from the random similarity matrix (M_r_). Similarly to the last step, the matrix sampling was performed with 2000 different numbers and repeated for 5000 times for each number. This sampling formed 10 million sub-matrices in total. For each matrix, the initial score and *Z* score of each column were calculated as previously described. And, the original score (*O*_r_) of random selected compounds was calculated. There were 10 million *O*_r_ values in total.

Because every original score (*O*) was calculated in the same length that was equal to the number of active compounds against the cell line, it was unnecessary to standardize this value. The significance of the original score, here defined as the association score (AS score, *P*_O_), was calculated as the probability of getting an random *O*_r_ that is not less than *O* score in random sampling.

$$P_{\text{o}}=N(O_{\text{r}}\geq O)/N(O_{\text{r}})$$ (7)

*N(O*_r_ *≥ O)* denotes the number of *O*_r_ which exceeds or equals *O*, and *N(O*_r_*)* indicates the total number of *O*_r_ (10 million). This final association score (AS or *P*_O_) reflects whether the protein has a function in proliferation, apoptosis, or differentiation of cancer cells.

Molecular property and similarity calculation and automatic data processing were performed with Pipeline Pilot (version 8.5). The TL-SEA algorithm was implemented with a MATLAB script (7.14, 2012a).

### Protein-compound association networks

During the process of AS score (*P*_O_) calculation, a *P*-value (*P*_Z_) matrix between the NCI compounds and the BindingDB proteins was formed. For matrix elements with very low *P*_Z_ value, there was a great chance that the corresponding NCI compound and the protein can bind to each other. To illustrate the relationships between NCI chemicals and BindingDB proteins clearly, we retained the matrix elements with *P*_Z_ lower than 0.0001 and converted the matrix into a chemical-protein interaction network. The network was graphically presented, rendered with Cytoscape [79] (version 2.8.2) by Force-Directed BioLayout. The edge was weighted by the reciprocal of the negative of the common logarithm of *P*_Z_.

## SUPPLEMENTARY MATERIAL FIGURES AND TABLES






