# Supplementary material for: Predicting cancer-relevant proteins using an improved molecular similarity ensemble approach
Source: Oncotarget. 2016 Apr 13;7(22):32394–407. doi: 10.18632/oncotarget.8716 (PMC5078021; doi:10.18632/oncotarget.8716)
Supplement: Supplementary file 1 [file oncotarget-07-32394-s001.pdf]

Predicting cancer-relevant proteins using an improved molecular similarity ensemble approach

Supplementary Materials

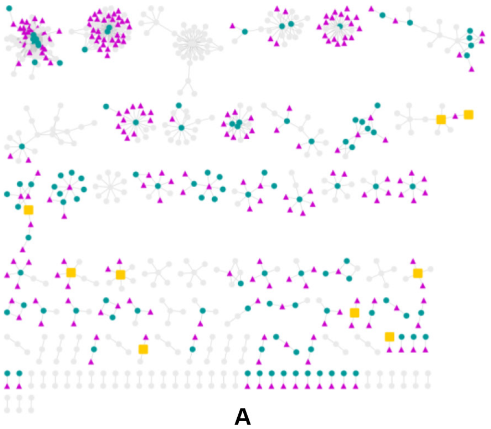

A

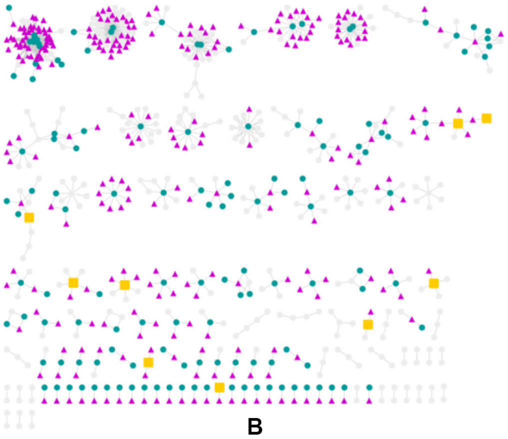

B

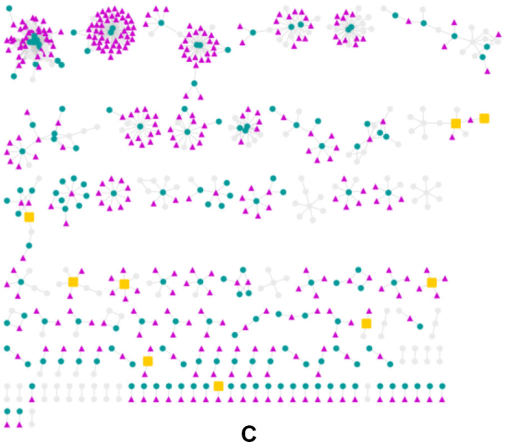

C

Supplementary Figure S1: The scattered network of chemical-protein association. The NCI compounds are represented with triangle nodes. Proteins are denoted with round nodes. Among the proteins, the important ones are denoted as orange squares. (**A**) Scattered network for blastic phase of chronic myelogenous leukemia (K562) cell line active compounds and proteins. Gray nodes denote that it does not appear in this system. (**B**) Scattered network for Non Small Cell Lung cancer (A549) cell line. (**C**) Scattered network for breast cancer (MCF7) cell line.

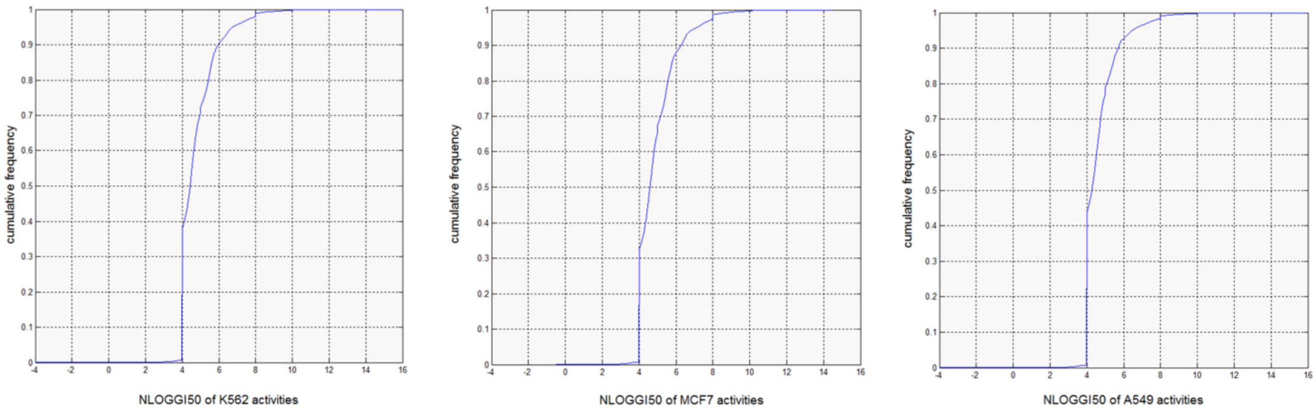

Supplementary Figure S2: Cumulative frequency of pGI$_{50}$ values for active compounds against the three cancer cell lines. Approximately 90% active compounds possessed the activity less than 6 (GI$_{50}$ $\geq 10^{-6}$ mol/L). Therefore, compounds with pGI50 over 6 were defined as active. Inactive compounds were discarded.

Molecular Weight

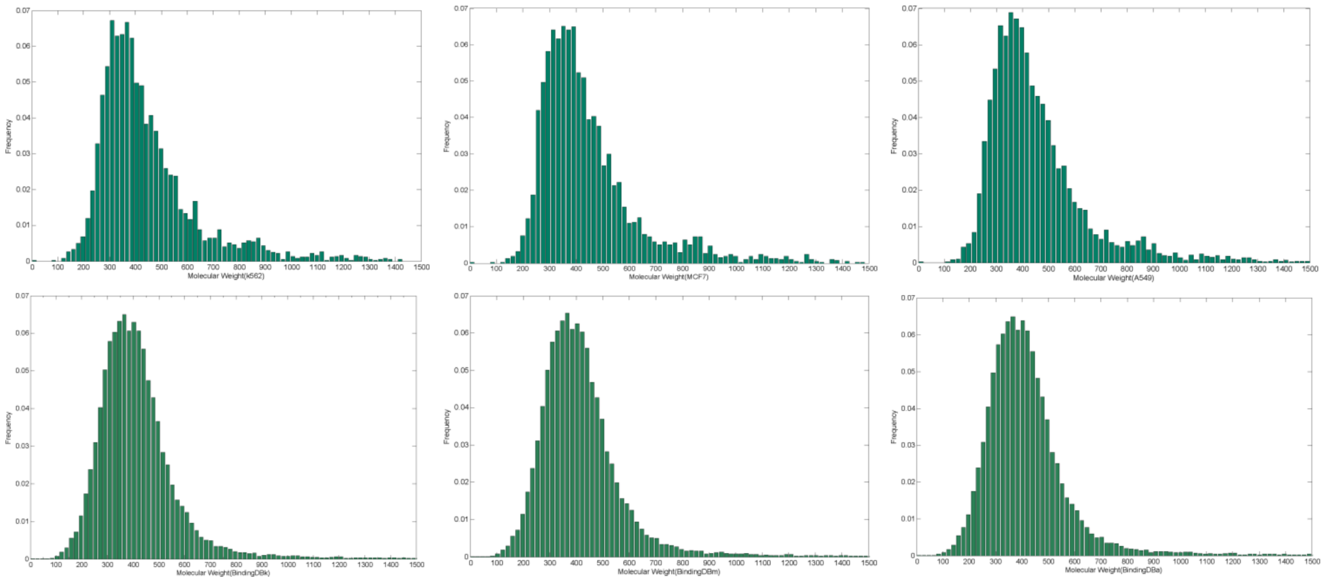

ALogP

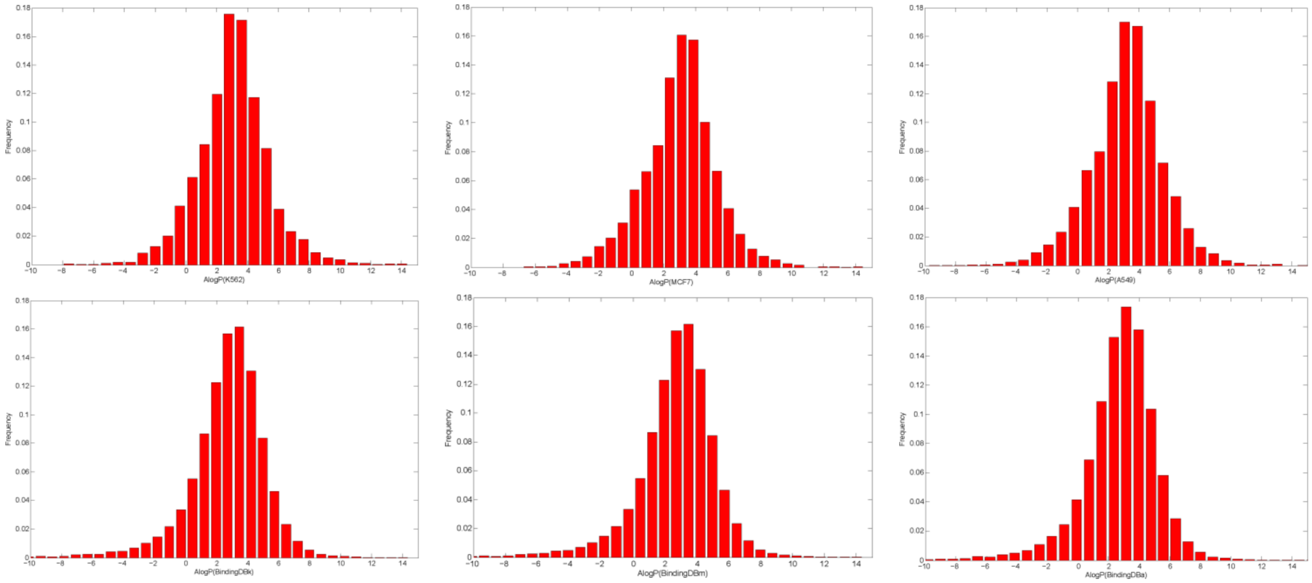

Supplementary Figure S3: The distribution of molecular weight (top, green) and ALogP (bottom, red) of the active compounds against the three cancer cell lines and three groups of random compounds in Binding DB. Property thresholds were set to [2, 7] & [150, 750] (for AlogP and molecular weight), [–3, 8] & [200, 800], [–3, 8] & [200, 800] for the three cell lines respectively.

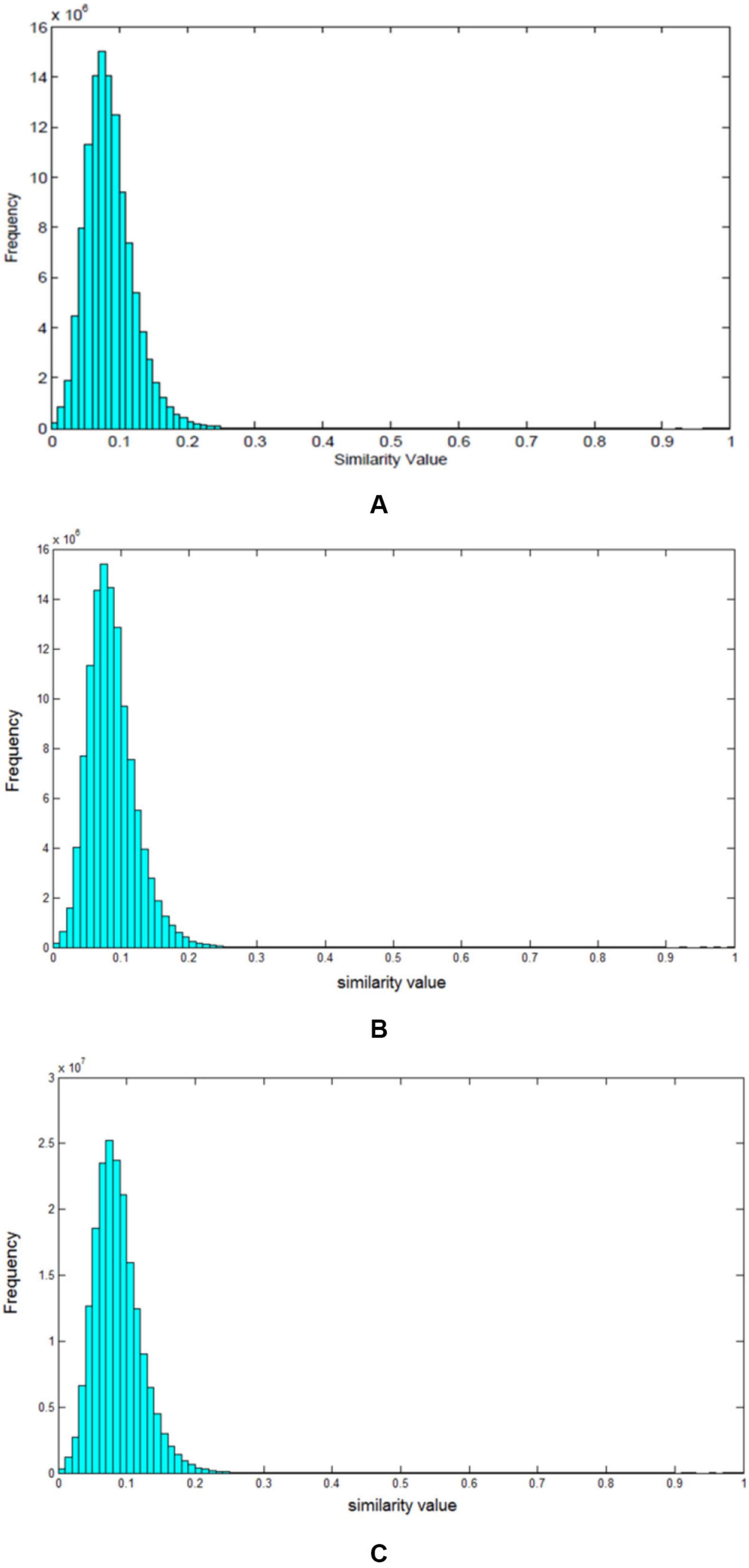

Supplementary Figure S4: The frequency histograms of the similarity between random compounds and active compounds against the three cancer cell lines, K562, MCF7, A549. About 5% of the similarity values are larger than 0.15. Therefore, the similarity threshold was set to 0.15 to distinguish significant similarity values from background noise.

Supplementary Table S1: Predicted interaction in the two sub-networks in Figure 4

Sub-network C'

Protein_ID	P_Z (Compound 727680)	Protein_ID	P_Z (Compound 727680)
P23097	1.85E-06	P34960	2.20E-05
P51511	1.59E-05	Q13443	3.52E-05
P51512	1.76E-05	P09237	4.70E-05
P17301	2.01E-05		

Sub-network C''

Chemical_ID	P_Z (protein P05227)	Chemical_ID	P_Z (protein P05227)
365360	4.65E-06	671553	4.42E-05
766057	6.65E-06	353	4.87E-05
69354	9.85E-06	8591	4.87E-05
743862	1.14E-05	56618	5.30E-05
762540	1.18E-05	1063	5.60E-05
762541	1.51E-05	17273	8.13E-05
29459	1.66E-05	13483	9.28E-05
695945	2.05E-05	695939	9.87E-05
762542	2.05E-05		

Supplementary Table S2: Rank list of P_Z ($P_Z < 0.0001$) in MCF7 dataset. See Supplementary_Table_S2

Supplementary Table S3: Similarity between active compounds against protein (P05227) and NCI compounds (4) in subnetwork of MCF7. See Supplementary_Table_S3
